# Decreased methylation of ZNF671 suppresses tumor progression by promoting MAPK6 transcription in laryngeal carcinoma

**DOI:** 10.7150/ijbs.82692

**Published:** 2023-05-08

**Authors:** Wei Zhang, Ting Liu, Xinyi Li, Tianshu Li, Xiangchi Ma, Dongxu Zhao, Xudong Zhao

**Affiliations:** 1 Department of Endocrinology, Shengjing Hospital of China Medical University, Shenyang, Liaoning, 110004, China.; 2 Department of Otolaryngology Head and Neck Surgery, Shengjing Hospital of China Medical University, Shenyang, Liaoning, 110004, China.

**Keywords:** Laryngeal carcinoma, ZNF671, MAPK6, methylation, proliferation

## Abstract

**Background:** Laryngeal squamous cell carcinoma (LSCC) is a malignant tumor of the head and neck, the exact mechanism of which has not been explored.

**Methods:** By analyzing the GEO data, we found the highly methylated and low expression gene ZNF671. The expression level of ZNF671 in clinical samples was verified by RT-PCR, western blotting and methylation-specific PCR. The function of ZNF671 in LSCC was detected by cell culture and transfection, MTT, Edu, TUNEL assays and flow cytometry analysis. The binding sites of ZNF671 to MAPK6 promoter region were detected and verified by luciferase reporter gene and chromatin immunoprecipitation. Finally, the effect of ZNF671 on LSCC tumors was tested *in vivo*.

**Results:** In this study, by analyzing GEO data GSE178218 and GSE59102, we found that zinc finger protein (ZNF671) expression was decreased, and DNA methylation level was increased in laryngeal cancer. Moreover, the abnormal expression of ZNF671 was associated with poor survival prognosis of patients. In addition, we found that overexpression of ZNF671 could inhibit the viability, proliferation, migration and invasion of LSCC cells, while promoting cell apoptosis. In contrast, the opposite effects were observed after knockdown of ZNF671. Through the prediction website and chromatin immunoprecipitation and luciferase reporter experiments, it was found that ZNF671 could bind to the promoter region of MAPK6, thereby inhibiting the expression of MPAK6.* In vivo* experiments confirmed that overexpression of ZNF671 could inhibit tumor growth.

**Conclusion:** Our study found that ZNF671 expression was down-regulated in LSCC. ZNF671 up-regulates the expression of MAPK6 by binding to its promoter region, thus participating in cell proliferation, migration and invasion in LSCC. Our study may provide new ideas for early prediction and treatment of LSCC.

## Introduction

Laryngeal carcinoma is a malignant tumor of the head and neck, with an increasing incidence and mortality in recent years 1. More than 90% of laryngeal cancers were confirmed as laryngeal squamous cell carcinoma (LSCC) by pathology 1. Despite improvements in surgical treatment of laryngeal cancer in the last 20 years, the five-year survival rate for laryngeal cancer is rather low 2, 3. At present, the most common treatment for laryngeal squamous cell carcinoma is total laryngectomy combined with postoperative radiotherapy 4. Since laryngectomy severely affects the laryngeal function of patients and leads to poor quality of life, it is extremely important to explore the exact molecular pathogenesis of LSCC. LSCC is the result of multiple complex factors, including environmental factors (smoking, alcohol consumption, air pollution and infection) and genetic factors 5. At the same time, increasing studies reported that there are many abnormal epigenetic modifications in LSCC 6. Cancer is driven by cumulative genetic and epigenetic alterations. CpG island hypermethylation is a common epigenetic change found in almost all types of human tumors 7, 8. Therefore, methylation may be an ideal method for early detection of tumors. For example, previous studies have found that promoter hypermethylation is associated with tumor suppressor gene silencing in breast cancer. 9.

Zinc-finger proteins (ZFPs) are currently the largest family of transcription factors, and their zinc-finger domains bind to gene promoters to activate or inhibit the expression of target genes 10. Nearly one third of ZFPs contain a highly conserved Kruppel-associated box (Krab) motif that promotes transcriptional repression by recruiting histone deacetylase complexes 10, 11. Previously identified Krab-ZFP proteins, including zinc finger protein (ZNF) 382, ZNF545, and ZNF331, are frequently silenced in cancer due to promoter hypermethylation 12. ZNF671 is a member of the Krab-ZFP family of mammalian transcriptional repressors, including C2H2-type zinc finger proteins and Kruppel-associated frame domain (KRAB) 13. ZNF671 recruits KRAB-related protein-1 and other co-repressors that have been involved in the pathological process of tumors by regulating cell differentiation, proliferation, and apoptosis 13. Increasing studies have shown that ZNF671, as a tumor suppressor, is epigenetically inhibited by DNA methylation in a variety of tumors 13-15. Sun et al. showed that overexpression of ZNF671 inhibited tumor growth by regulating cell cycle and delaying cell cycle progression 16, 17. Kondo et al published studies showing that ZNF671 with high DNA methylation status may be an effective biomarker for predicting recurrence of ovarian cancer 18. However, the function and role of ZNF671 in LSCC have not been studied.

In our study, by analyzing GEO data GSE178218 and GSE59102, we found that ZNF671 expression was decreased, and its DNA methylation level was increased in LSCC, and abnormal expression of ZNF671 was associated with poor survival prognosis of patients. Functional experiments were conducted to verify the role of ZNF671 ifn the progression of LSCC and the mechanism of ZNF671. Our study may provide new insight for early prediction and treatment of LSCC.

## Methods

### Bioinformatics analysis

Two groups of data of normal group and LSCC tissue were analyzed based on GSE178218 and GSE59102 data. GSE178218 included 11 non-tumor adjacent tissues and 20 tumors from LSCC patients, and GSE59102 included 29 LSCC tissue and 13 para-carcinoma tissue. The expression levels of ZNF671/MAPK16 in head and neck cancer (TCGA-HNSC) were analyzed by UALCAN and GEPIA based on TCGA database. GEPIA and Kaplan-Meier Plotter were performed to analyze the effect of ZNF671/MAPK16 expression on prognosis. In addition, the online tool AnimalTFDB (v3.0) was used to analyze the binding promoter TFBS (TF binding site) of transcription factor ZNF671, and it was found that MAPK6 and SIAH1 could bind ZNF671 in the protein interaction network. UALCAN and GEPIA was used according to the following methods. Open the UALCAN (http://ualcan.path.uab.edu/index.html) or GEPIA (http://gepia.cancer-pku.cn/) database, select CPTAC analysis and tumor type, and then select total protein and survival.

### Clinical samples

Our study was approved by the Ethics Committee of Shengjing Hospital of China Medical University, and patients signed informed consent. 97 pairs of primary laryngeal carcinoma tissue samples were obtained from Shengjing Hospital of China Medical University, all of which were surgically resected and radical resection of LSCC. Samples taken during surgery were stored partly in liquid nitrogen and partly in 4% paraformaldehyde for subsequent detecting of gene and protein levels. None of the patients received preoperative chemoradiotherapy, and all of them were confirmed as laryngeal squamous cell carcinoma by pathology.

### Cell culture and transfection

Human laryngeal epithelial cells (ULA-TVC), LSCC lines (AMC-HN-8, TU686, TU177) and 293T were purchased from ATCC Cell Resource Center, USA. AMC-HN-8 and TU177 cells were cultured in RPMI 1640 medium containing 10% fetal bovine serum (FBS) with 5% CO_2_ at 37 °C. AMC-HN-8/TU177 cells (5×10^5^ cells/mL) were seeded in 6-well plate. When the cells grew to 70% confluence, shZNF671, pcDNA-ZNF671 and their respective negative controls were transfected into cells using Lipofectamine 2000 (Invitrogen, Carlsbad, CA, USA). shZNF671, pcDNA-ZNF671 were constructed from Genechem Co. (Shanghai, China).

### Immunohistochemistry

After operation, paraffin embedded LSCC tissue and adjacent tissue sections (4 μm) were removed and dewaxed with xylene, absolute ethanol, phosphate buffered saline (PBS), and 3% hydrogen peroxide was used to inactivate endogenous peroxidase 19. Then the sections were removed and washed with PBS. The antigen was repaired by thermal repair method. And then the sections were removed and blocked for 10 min. The specific primary antibody was added and incubated at 4°C for 12 h. Specific primary antibodies were as follows: ZNF671 (Sigma-Aldrich, Shanghai, China; HPA046099, 1:100), MAPK6 (Abcam, Cambridge, MA, USA; ab53277, 1:100), Ki67 (Abcam, ab15580, 1:100), E-cadherin (Abcam, ab76055, 1:100), N-cadherin (Abcam, ab76011, 1:100). After incubation, the cells were washed, and then biotin-labeled secondary antibody and horseradish peroxidase labeled chain enzyme ovalbumin were added successively, and the cells were incubated at 4°C for 20 min. After incubation, the sections were washed again and stained with chromodevelopment solution for 5 min. After washing, counterstaining, dehydration and sealing, the sections were placed under a microscope for observation. Five high-power fields were randomly selected from each section and the number of positive staining cells was counted. The reagents used in this experiment were purchased from Wuhan Goodbio Technology Company.

### RT-PCR

Total RNA was extracted from LSCC cells or tissue using TRIzol reagent (Takara, Dalian, China), and cDNA was synthesized according to the instructions of reverse transcription kit (Takara) 20. Specific primers were used to amplify cDNA as template. Specific primers were as follows: ZNF671 5'-GGTCCTATGGCGGAGCTAAC-3' and 5'-CATGGTACAAAAGTCTCTGAGCA-3', MAPK6 5'-TGTCAAACATGCTCTACGTGAAA-3' and 5'-TCGTCTGTTAATTGGCTTCCAC-3', SIAH1 5'-AGCCGTCAGACTGCTACAG-3' and 5'-AAAAGACTCGCCAAGTCATTGT-3'; GAPDH 5'-TCAAGGCTGAGAACGGGAAG-3' and 5'‐TGGACTCCACGACGTACTCA-3'. According to Ct values, the expression of target genes was calculated by 2^-ΔΔCT^ method.

### Western blotting

Tissue or cells were collected, and RIPA lysis buffer was added to extract cell proteins on ice. The 30 μg protein sample was electrophoresed by 10% sodium dodecyl sulfate and polyacrylamide gel electrophoresis, and then transferred into PVDF membrane 21, 22. The membranes were blocked with 5% skim milk powder for 1 h, and the specific primary antibodies (1:1000 dilution) were added and incubated at 4°C overnight. Specific primary antibodies were as follows: ZNF671 (Proteintech, Chicago, IL, USA; 21329-1-AP), cyclin D1 (Abcam, ab16663), CDK4 (Abcam, ab108357), bcl-2 (Abcam, ab32124), cleaved caspase-3 (Abcam, ab32042), E-cadherin (Abcam, ab76055), N-cadherin (Abcam, ab76011), SIAH1s (Abcam, ab2237), MMP-2 (Abcam, ab92536), and MMP-9 (Abcam, ab76003). The PVDF membrane was washed with TBST solution for 10 min, three times, developed with chemiluminescence solution, and the protein gray value was analyzed by Image J software. β-actin was used as the reference protein to calculate the target protein expression.

### Cell proliferation was detected by MTT assay

AMC-HN-8/TU177 cells were seeded in 96-well plate at a density of 1×10^4^/mL and cultured for 24 h. The cells were treated according to different experimental requirements. The treated cells were cultured for 48 h, then 20 μl of 0.5% MTT solution (Invitrogen) was added for 4 h, and 150 μl of dimethyl sulfoxide (Invitrogen) was added for 10 min, as previously stated 23, 24. The absorbance (OD) value of LSCC cells was measured by microplate analyzer.

### Methylation specific PCR

DNA was extracted from tissues or cells and quantified using a UV spectrophotometer. Then, 1 μg of DNA was modified according to the instructions of the genomic DNA modification kit. The modified DNA was used as a template for PCR amplification using methylated and non-methylated specific primers. Finally, agarose gel electrophoresis was performed, and 20 μl of the methylation specific PCR amplification products were taken for 2% agarose gel electrophoresis. The bands were observed under UV light, and then gel imaging was performed, and the results were analyzed.

### 5-Ethynyl-2-deoxyuridine (Edu) staining

To detect the proliferation of LSCC cells, we added 50 μM Edu (Invitrogen) to the treated cells, and then continued to culture the cells for 2 h. Cells were washed with PBS, fixed by adding 50 μl of 4% paraformaldehyde containing 0.5% Triton X-100 for 0.5 h, and then neutralized with 2 mg/mL glycine solution. After washing the cells with PBS, 100 μl of 1× Apollo dye was added and left at room temperature for half an hour. Finally, add 100 μl of Hoechst 33342 (Invitrogen) to each well and leave for another half hour. Edu positive cells were observed by microscope and analyzed statistically.

### TUNEL assay

The cells were digested with trypsin, then RPMI 1640 medium containing 10% FBS was added to neutralize the trypsin, and the suspended cells were blown, the cell suspension was counted, and the cell concentration was adjusted to 1×10^4^/mL. The above cell suspension was seeded in a 6-well plate with crawling slices inserted, with 500 μl in each well. The apoptotic cells were observed under fluorescence microscope. When calculating the results, 10 high magnification fields were randomly selected for each sample, and the proportion of TUNEL-positive cells in all cells was counted to reflect the apoptosis rate of tumor cells.

### Flow Cytometry

The treated cells were digested with trypsin and the cell suspension was collected, added with 1 mL 75% precooled ethanol, blown evenly, fixed at 4°C for more than 12 h, and then washed with PBS twice, and centrifuged at 1000 r/min for 5 min. The cells were resuspended in 0.5 mL PBS, and propidium iodide (Biolegend, San Diego, CA, USA) and RNaseA were added to each well until the final concentration was 50 μg/mL in 37°C warm bath for 30 min. Cell cycle was detected by flow cytometry.

### Scratch assay

LSCC cells were cultured in 6-well culture plate after trypsin digestion, and the cells were treated according to different experimental requirements. When the cell confluence reached about 90%, the sterilized 200 μl spearhead was used to make scratches along a straight line in the center of the bottom of each well, so as to keep the scratch width of the cells in each well consistent. After washed with PBS, the cell debris caused by scratches was gently removed, and the culture medium was added with serum-free medium for 24 h. The scratch width at 0 h and 24 h was recorded in a microscope, and the migration rate was calculated.

### Transwell assays

To test the migration ability of LSCC cells, AMC-HN-8/TU177 cells were incubated with FBS-free RPMI 1640 medium at a density of 2×10^4^ cells /mL. 100 μl of cell suspension was added to the upper chamber of transwell, and 500 μl of RPMI 1640 medium containing serum was added to the lower chamber. Excess cells were removed by sterile cotton swab, fixed with 4% formaldehyde, and stained with 0.1% crystal violet to record the number of cell migration. To investigate the invasiveness of LSCC cells, matrigels (Beyotime, Guangzhou, China) were diluted with FBS-free RPMI 1640 medium at a ratio of 1:5, and 50 μl was absorbed into the upper chamber and allowed to stand for 3-4 h. The other operations were the same as LSCC cell migration ability.

### Luciferase reporter assay and ChIP

In order to explore the mechanism of ZNF671 on MAPK6, the MAPK6 promoter sequence was prepared into reporter gene plasmid and co-transfected with ZNF671 overexpression or knockdown plasmid into AMC-HN-8 and TU177 cells. Luciferase reporter assay was performed to detect the luciferase activity of MAPK6 promoter. In order to determine the specific site of ZNF671 binding to MAPK6 promoter, the 2000bp sequence of MAPK6 promoter was divided into 4 segments (0, -500, -1000, -1500, -2000) to make a reporter plasmid, which was co-transfected with ZNF671 expression plasmid into AMC-HN-8 and TU177 cells. Luciferase reporter assay was performed to detect the luciferase activity of MAPK6 promoter at different locations.

Immunoprecipitation was performed with anti-ZNF671 or anti-IgG, and protein A/G magnetic beads were used to collect the immunoprecipitation complexes. After that, PCR and agarose gel electrophoresis were used to purify and identify the DNA fragments. ChIP assays were performed with 293T cells, as previously reported. The bound DNA fragments were quantified by standard PCR and real-time PCR.

### *In vivo* experiment

Animal experiments in this study were approved by the Ethics Committee of Shengjing Hospital of China Medical University. We purchased SPF-grade male BALB/c nude mice from Beijing Vital River Laboratory Animal Technology, which were 6-8 weeks old. After overexpression or knockdown of ZNF671 in AMC-HN-8/TU177 cells, 2 × 10^6^ cells were suspended in 200 μl FBS-free DMEM, and LSCC cell suspension was subcutaneously inoculated in the right abdomen of nude mice. Tumor volume was measured from 8 days after injection. After 24 days, the mice were sacrificed under anesthesia. Tumors were dissected, weighed, and histologically analyzed.

### Statistical analysis

Graphpad 7.0 software was used for statistical analysis. Student's t test was used for comparison between groups, differences between multiple groups were used one-way ANOVA and Kaplan-Meier was used to analyze survival ratio. Data shown are represented as mean ± SD.* P* < 0.05 was considered statistically significant.

## Results

### Differentially expressed genes (DEGs) and DNA methylation analysis in LSCC based on GEO microarray data

**Figure 1:**
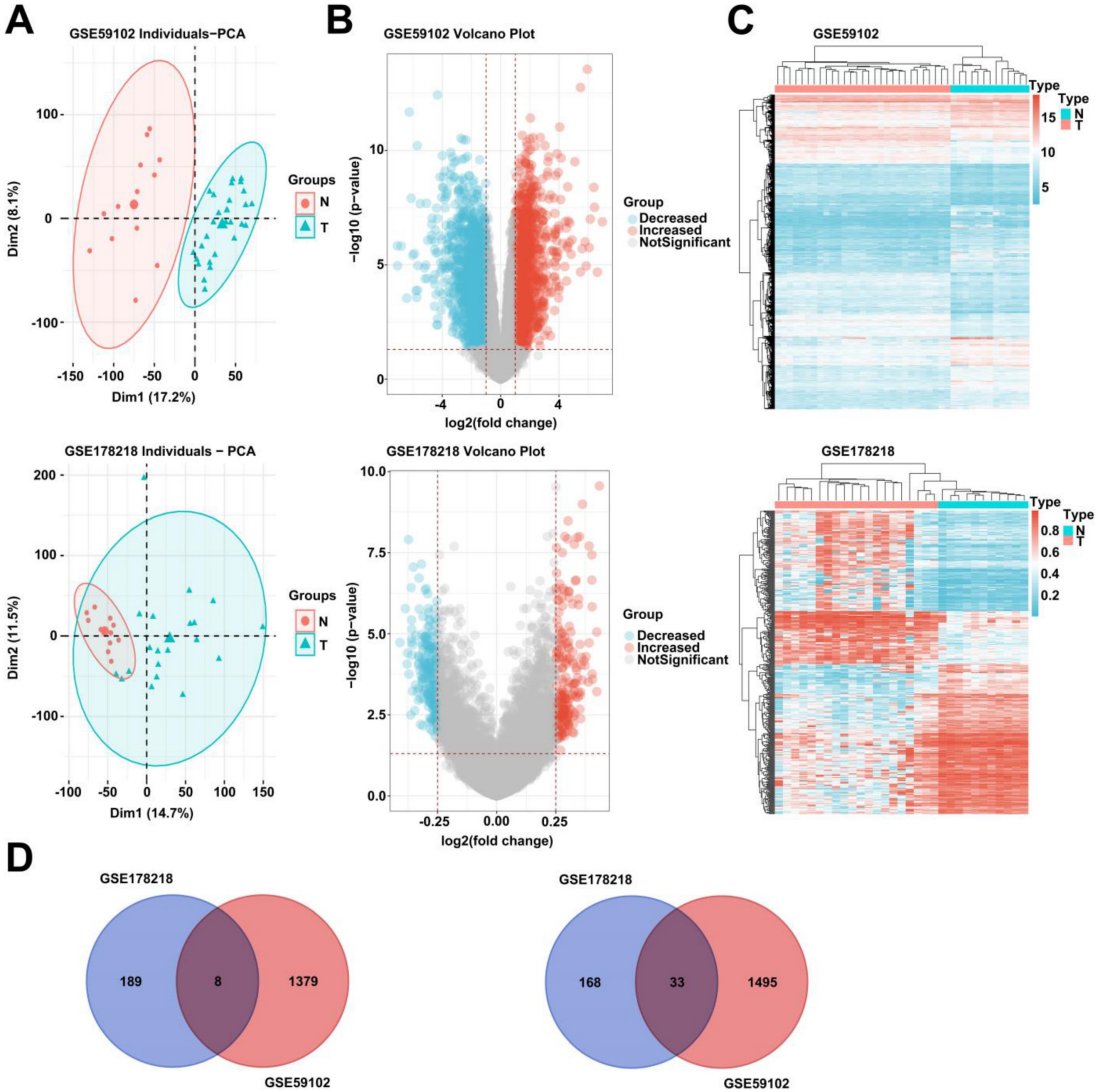
** Differentially expressed genes and DNA methylation analysis in LSCC based on GEO microarray data.** (A) Two groups of data of normal group and LSCC tissue were analyzed based on GSE178218 and GSE59102 data, and PCA (A), volcano map (B) and heat map (C) were drawn. (D) The intersection of genes up-regulated by DNA methylation and genes with decreased expression was selected (left), and the intersection of genes down-regulated by DNA methylation and genes with increased expression was selected (right).

To explore the possible pathogenic mechanism of LSCC, we analyzed the differentially expressed genes and DNA methylation levels of LSCC in GEO database (GSE59102 and GSE178218). After adjusting the data, principal component analysis (PCA) showed that the tumor group and the normal group were clustered in the two databases (Fig. 1, A). In addition, we analyzed GSE59102 data and obtained 1387 up-regulated and 1528 down-regulated genes among differentially expressed genes (Fig. 1, B). GSE178218 data obtained 201 up-regulated and 197 down-regulated genes with methylation levels among differentially expressed genes (Fig. 1, B). Further, we conducted cluster analysis on the two data chips, and we found that the data of GSE178218 and GSE59102 were significantly clustered (Fig. 1, C). Finally, we selected the intersection of genes with up-regulated DNA methylation in GSE178218 data and down-regulated DEGs in GSE59102, and the genes with down-regulated DNA methylation of GSE178218 data and increased DEGs in GSE59102. Eight hypermethylated and 33 hypermethylated and under expressed genes were obtained (Fig. 1, D).

### ZNF671 is decreased and hypermethylated in LSCC

**Figure 2:**
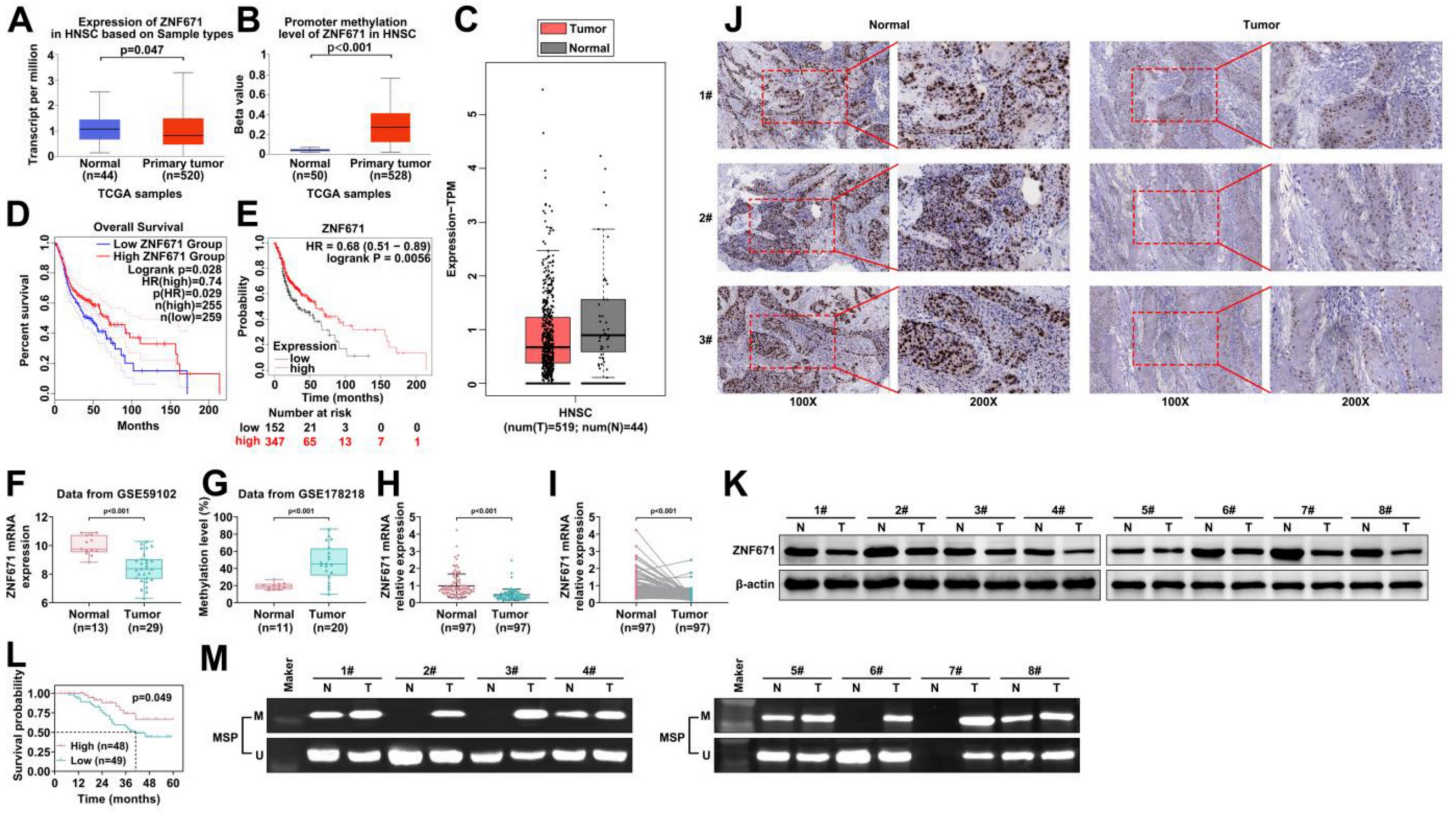
** ZNF671 is decreased and hypermethylated in LSCC.** (A-B) The expression levels (A and C) and promoter methylation levels (B) of ZNF671 in head and neck cancer (TCGA-HNSC) were analyzed using online platforms UALCAN and GEPIA based on TCGA database. (D-E) GEPIA (D) and Kaplan-Meier (E) were used to analyze the relationship between ZNF671 expression and the prognosis of LSCC. (F-G) Gene expression levels of ZNF671 in GSE59102 and GSE178218 microarrays. (H-I) Relative mRNA expression level of ZNF671 in normal group and LSCC tissues was detected by RT-PCR. (J) Representative immunohistochemical images of ZNF671 are in the normal group and the LSCC group. (K) Expression of ZNF671 protein in normal group and LSCC group. (L) Survival rate of LSCC patients in high and low ZNF671 groups. (M) Methylation specific PCR was used to detect the methylation level of ZNF671 in tumor tissues and control tissues.

DNA methylation is the first epigenetic modification discovered 25. Studies have revealed that DNA methylation is involved in the pathogenesis of a variety of tumors and is closely related to tumor treatment 26, 27. Among the 33 genes analyzed above, we focused on ZNF671. Previous studies have shown that the hypermethylation level of ZNF671 is closely related to serous recurrence of ovarian cancer 18. The expression level of ZNF671 in TCGA-HNSC was analyzed using online platforms UALCAN and GEPIA based on TCGA database. The results showed that the expression level of ZNF671 was decreased, and the methylation level was increased in HNSC (Fig. 2, A-C). In addition, GEPIA and Kaplan-Meier plotter were used to analyze the effect of ZNF671 expression on prognosis, and the results suggested that patients with low ZNF671 expression had poor prognosis (Fig. 2, D and E). We also analyzed the expression level of ZNF671 in GSE59102 and GSE178218, and the results showed that the expression level of ZNF671 gene was significantly decreased, and the methylation level was significantly increased in the tumor group (Fig. 2, F and G). Our clinical samples further confirmed that ZNF671 gene expression was significantly down-regulated in LSCC (Fig. 2, H and I). Consistent with the gene expression level, the protein level of ZNF671 in LSCC was significantly down-regulated by immunohistochemistry and western blotting (Fig. 2, J and K). Then, we divided LSCC patients into high and low ZNF671 groups based on the expression level of ZNF671, and found that patients with LSCC in the low ZNF671 group had a poor prognosis (Fig. 2, L). To investigate the methylation level of ZNF671, methylation specific PCR was used to detect the methylation level of ZNF671 in tumor tissues and control tissues, and the results showed that ZNF671 was significantly hypermethylated in the tumor group (Fig. 2, M). These results suggested that ZNF671 is decreased and hypermethylated in LSCC, and the decreased ZNF671 level is associated with the poor prognosis of LSCC patients.

### ZNF671 inhibited the growth, migration and invasion of LSCC cells

**Figure 3:**
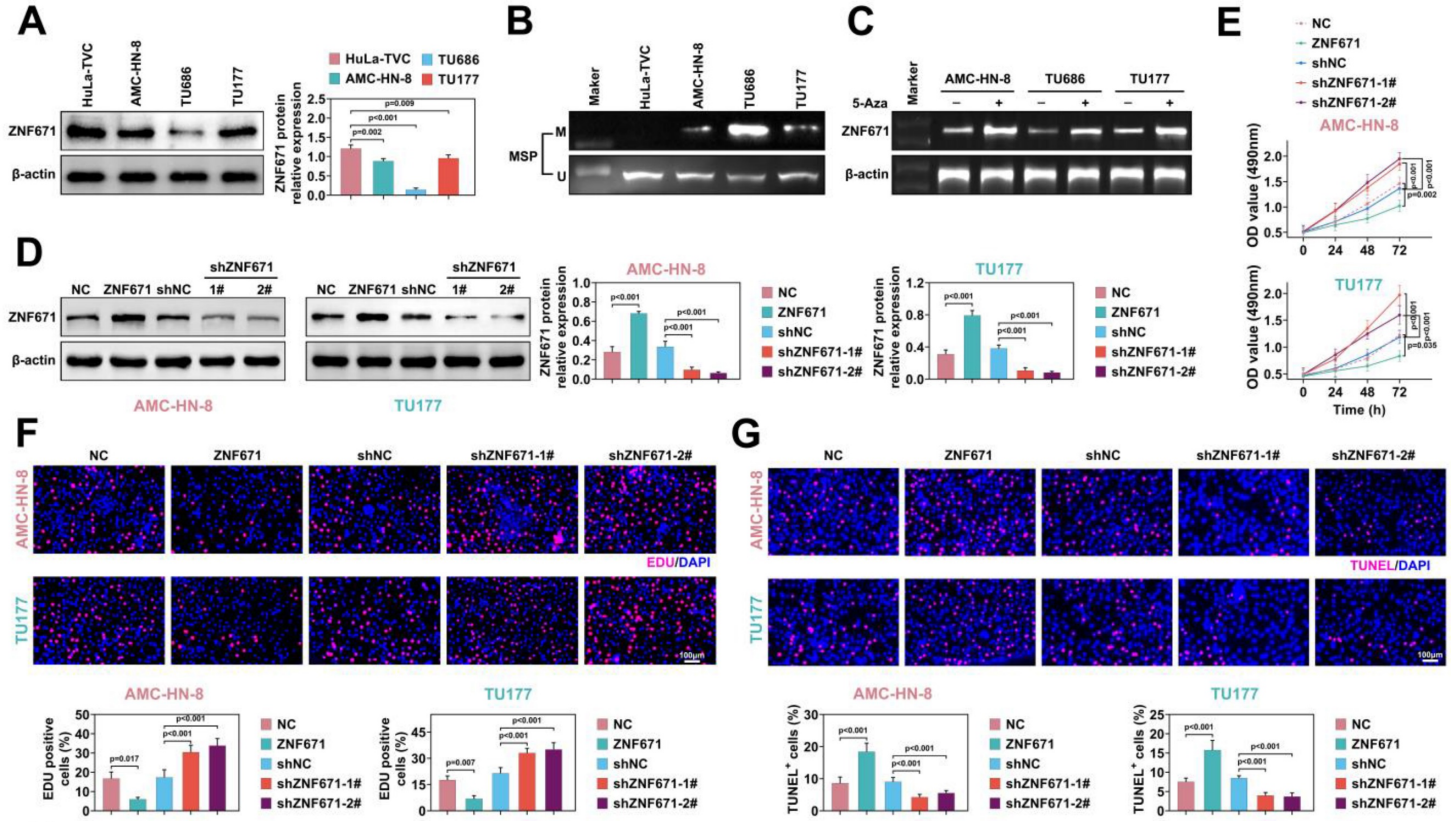

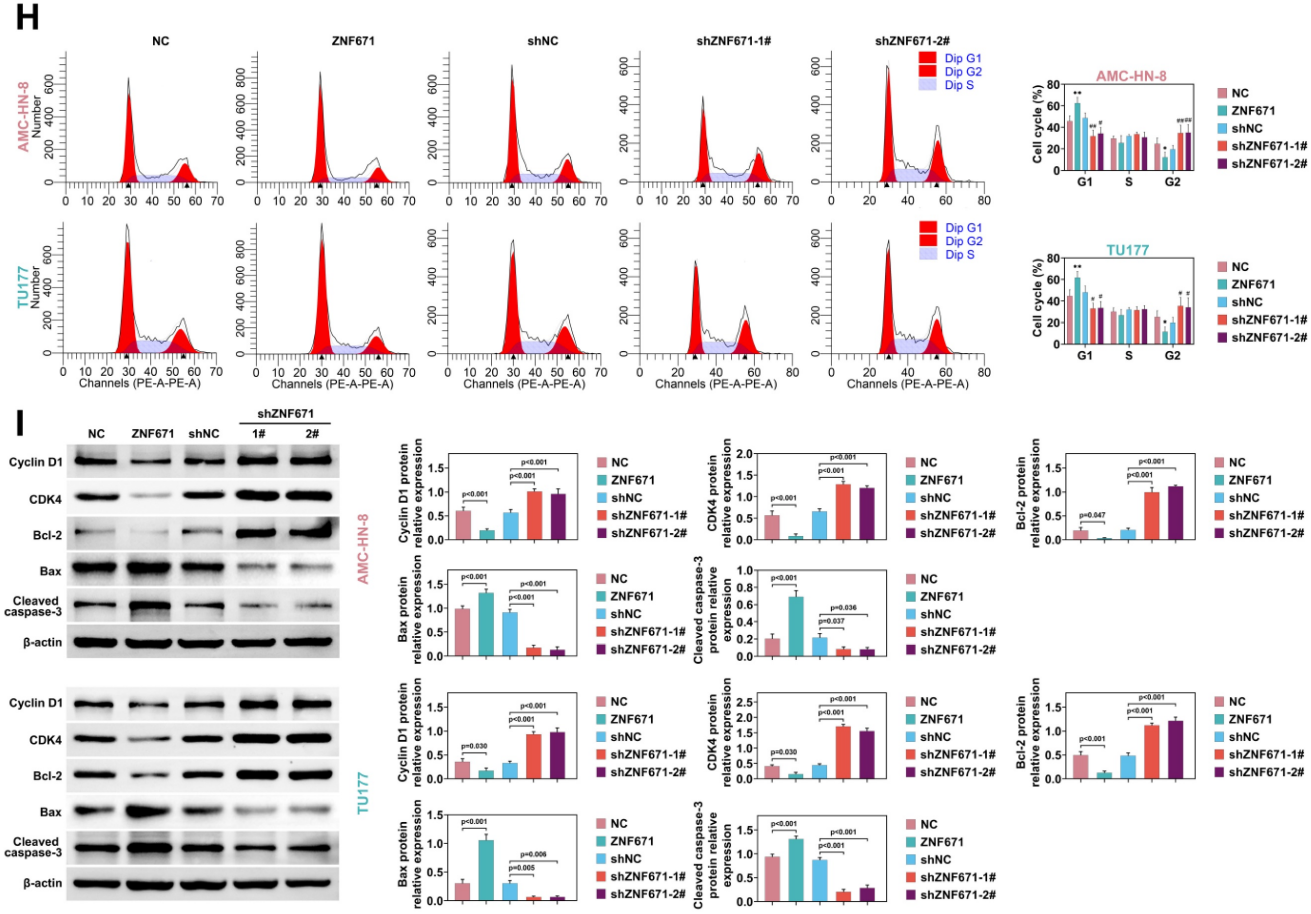
** ZNF671 inhibited the growth of LSCC cells.** (A-B) Expression levels (A) and methylation levels (B) of ZNF671 in human laryngeal epithelial cells (ULA -TVC) and LSCC lines AMC-HN-8, TU686, TU177. (C) mRNA level of ZNF671 was detected by PCR combined with gel electrophoresis in the presence/absence of 5-AZA (5-AZacytidine, a methyltransferase inhibitor). (D-I) ZNF671 was overexpressed or knocked down in AMC-HN-8 and TU177 cells. The protein level of ZNF671 was detected by western blotting (D), cell viability was detected by MTT (E), cell proliferation was detected by Edu (F), and apoptosis was detected by tunel (G). Flow cytometry was used to detect the cell cycle (H), and western blotting was used to detect the protein levels of cell cycle and apoptosis related indicators (I).

To discover the role of ZNF671 in LSCC, we selected LSCC cell lines. Compared with human laryngeal epithelial cells (ULA-TVC), the expression level of ZNF671 was lower in LSCC lines (AMC-HN-8, TU686 and TU177) (Fig. 3, A). Moreover, ZNF671 showed higher methylation levels in LSCC cell lines (Fig. 3, B). ZNF671 mRNA level was detected by PCR combined with gel electrophoresis in the presence/absence of 5-AZA (5-AZacytidine, a methyltransferase inhibitor). The results demonstrated that the methylation level of ZNF671 was markedly up-regulated in the presence of 5-AZA. To reveal the pathological mechanism of ZNF671 in LSCC, we overexpressed or down-regulated ZNF671 in AMC-HN-8 and TU177 cells and detected the expression of ZNF671 by western blotting (Fig. 3, D). We found that the cell viability was significantly weakened after overexpression of ZNF671, while it was significantly enhanced after knockdown of ZNF671 (Fig. 3, E). Then, the effect of ZNF671 on the proliferation of LSCC cells was detected by Edu assay. We found that the Edu positive cells were clearly decreased when ZNF671 was overexpressed, while the Edu positive cells were significantly increased when ZNF671 was knocked down, indicating that ZNF671 significantly affected the proliferation of LSCC cells (Fig. 3F). At the same time, the effect of ZNF671 on apoptosis of LSCC cells was detected by TUNEL assay. The results showed that when ZNF671 was overexpressed, TUNEL-positive cells were significantly increased while when ZNF671 was knocked down, tunel-positive cells were significantly decreased (Fig. 3, G), indicating that ZNF671 promoted apoptosis of LSCC cells. Furthermore, the effect of ZNF671 on LSCC cell cycle was investigated by flow cytometry. It was found that when ZNF671 was overexpressed, the cells in G1 phase were increased and the cells in G2 phase were significantly decreased, while when ZNF671 was knocked down, the cells in G1 phase were significantly decreased and the cells in G2 phase were significantly increased. This indicated that ZNF671 inhibited cell division (Fig. 3, H). Finally, we detected the levels of proteins related to cell cycle (cyclin D1, CDK4) and anti-apoptosis (bcl-2) by western blotting 28-31, and found that cyclin D1, CDK4 and bcl-2 were significantly down-regulated in LSCC cells after overexpression of ZNF671. Bax and cleaved caspase-3 were significantly up-regulated. However, the opposite result was found after knockdown of ZNF671 (Fig. 3, I).

**Figure 4:**
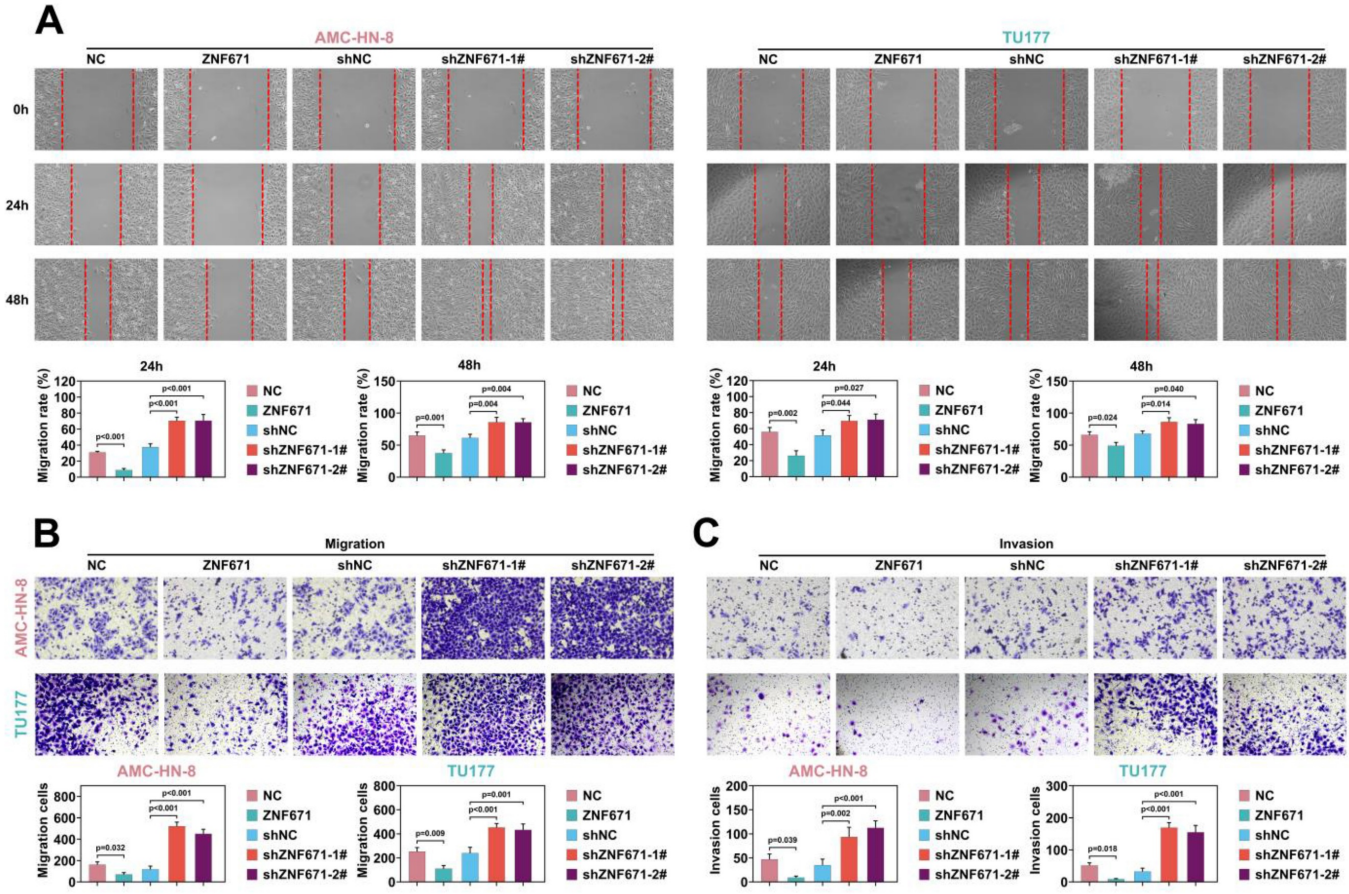

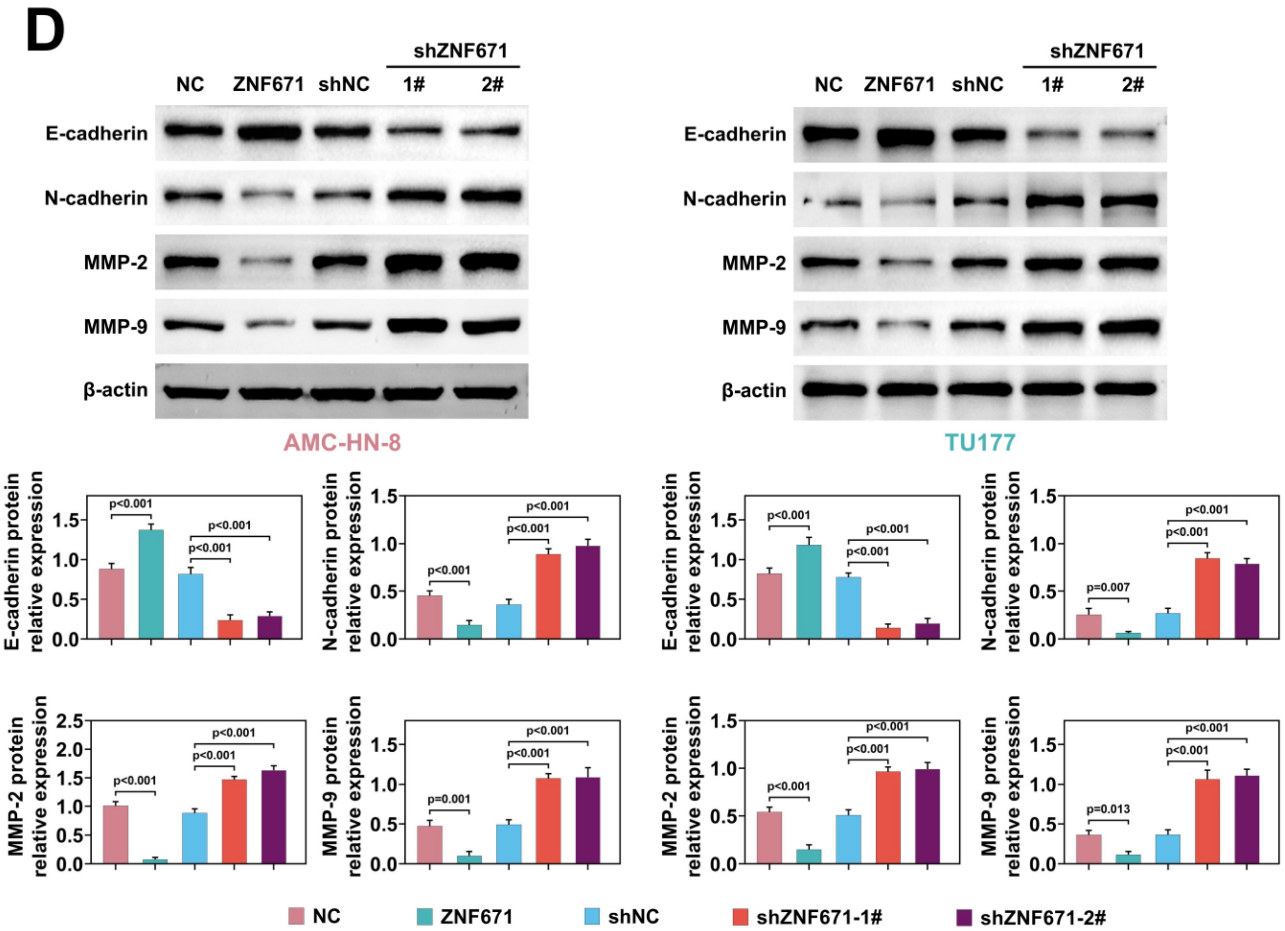
** ZNF671 inhibited migration and invasion of LSCC cells.** (A-D) ZNF671 was overexpressed or knocked down in AMC-HN-8 and TU177 cells. Cell migration was detected by scratch (A), cell migration (B) and invasion (C) were detected by transwell, and migration related protein levels were detected by western blotting (D).

Cell migration and invasion play a crucial role in LSCC 23, 32-34. Through scratch experiment, we found that the width of scratch was significantly widened when ZNF671 was overexpressed, and significantly narrowed when ZNF671 was knocked down (Fig. 4, A), indicating that ZNF671 significantly inhibited the migration of LSCC cells. Furthermore, transwell assay showed that after overexpression of ZNF671, the migrated and invaded cells were decreased, while after knockdown of ZNF671, the migrated and invaded cells were increased (Fig. 4, B and C), indicating that ZNF671 significantly affected the migration and invasion of LSCC cells. Finally, western blotting analysis showed that when ZNF671 was highly expressed, E-cadherin expression was increased, while N-cadherin, MMP-2 and MMP-9 expression were all decreased. However, when ZNF671 was knocked down, the opposite result was found (Fig. 4, D). All these results indicated that ZNF671 was related to the proliferation and invasion of LSCC cells.

### ZNF671 inhibited MAPK6 transcription in LSCC cells

**Figure 5:**
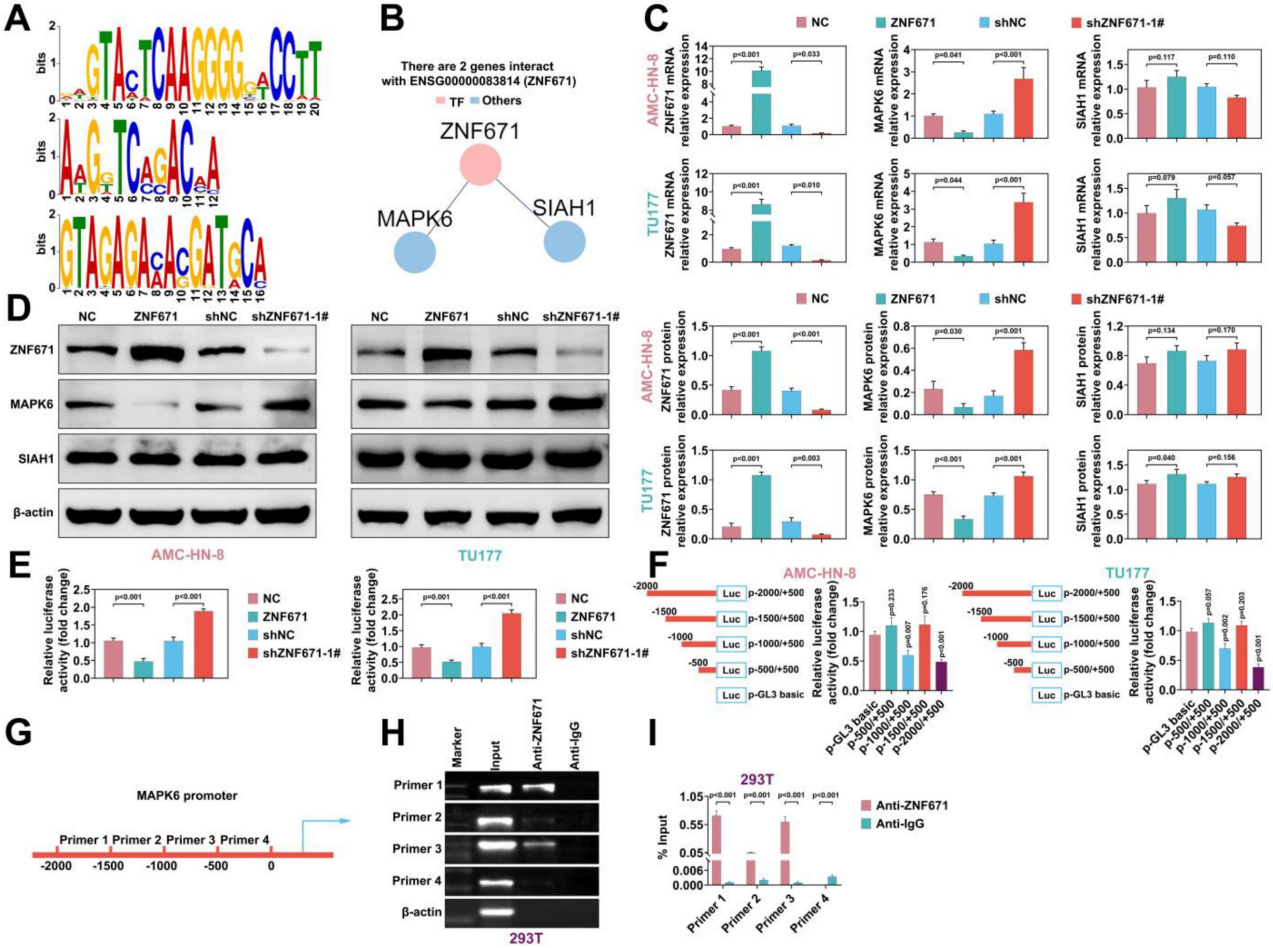
** ZNF671 inhibited MAPK6 expression in LSCC cells.** (A-B) The online tool AnimalTFDB (V3.0) was used to analyze the binding promoter TFBS (TF binding site) of transcription factor ZNF671 (A), and it was found that MAPK6 and SIAH1 could bind ZNF671 in the protein interaction network (B). (C-E) overexpressed or knocked down ZNF671 in AMC-HN-8 and TU177 cells, and the gene expression levels of ZNF671, MAPK6 and SIAH1 were detected by RT-PCR (C). The protein expression levels of ZNF671, MAPK6 and SIAH1 were detected by western blotting (D), and the luciferase activity of MAPK6 promoter was detected by luciferase reporter assay (E). (F) The 2000bp sequence of MAPK6 promoter was divided into 4 segments (0, -500, -1000, -1500, -2000) to make a reporter plasmid, which was co-transfected with ZNF671 expression plasmid into AMC-HN-8 and TU177 cells, and luciferase reporter assay was performed. The luciferase activity of MAPK6 promoter at different positions was detected. (G) Design primer sequence of MAPK6 promoter region. (H-I) The expression levels of MAPK6 in these four sequences were detected by CHIP-qPCR and CHIP-RT-PCR.

As a transcription factor, ZNF671 is speculated to bind to the promoter regions of certain genes 18. The online tool AnimalTFDB (v3.0) was used to analyze the binding promoter TFBS (TF binding site) of transcription factor ZNF671, and it was found that MAPK6 and SIAH1 could bind to ZNF671 in the protein interaction network (Fig. 5, A and B). To investigate the regulation of downstream genes of ZNF671, we overexpressed or down-regulated ZNF671 in AMC-HN-8 and TU177 cells. RT-PCR was performed to detect the efficiency of ZNF671 overexpression or knockdown, and the expression levels of MAPK6 and SIAH1. The results unfolded that overexpression of ZNF671 down-regulated the expression of MAPK6, while knockdown of ZNF671 up-regulated the expression of MAPK6. Neither overexpression nor knockdown of ZNF671 affected the expression level of SIAH1 (Fig. 5, C). Consistent with the gene level, the protein levels of ZNF671, MAPK6 and SIAH1 were confirmed by western blotting (Fig. 5, D). The MAPK6 promoter sequence was inserted into reporter gene plasmid and co-transfected with ZNF671 overexpression or knockdown plasmid into AMC-HN-8 and TU177 cells. Luciferase reporter assay was performed to detect the luciferase activity of MAPK6 promoter. The results showed that luciferase activity was decreased when ZNF671 was overexpressed and enhanced when ZNF671 was knocked down (Fig. 5, E). These results manifested that ZNF671 could down-regulate the expression of MAPK6. Further, in order to explore the specific site of ZNF671 action on MAPK6, the 2000bp sequence of MAPK6 promoter was divided into 4 segments (0, -500, -1000, -1500, -2000) to make a reporter plasmid, which was co-transfected with ZNF671 expression plasmid into AMC-HN-8 and TU177 cells. Luciferase reporter assay was performed to detect the luciferase activity of MAPK6 promoter at different locations. The results showed that promoter activity changed significantly at -2000 - -1500 and -1000 - -500 segments (Fig. 5, F). Further, the effect of ZNF671 on the transcription of MAPK6 promoter region was analyzed by ChIP-RT-PCR assay, and it was found that primer 1 and primer 3 segments were significantly enriched (Fig. 5, G and H). Moreover, ZNF671 significantly affected the transcription of these MAPK6 promoter regions (Fig. 5, I).

### MAPK6 was upregulated in LSCC

**Figure 6:**
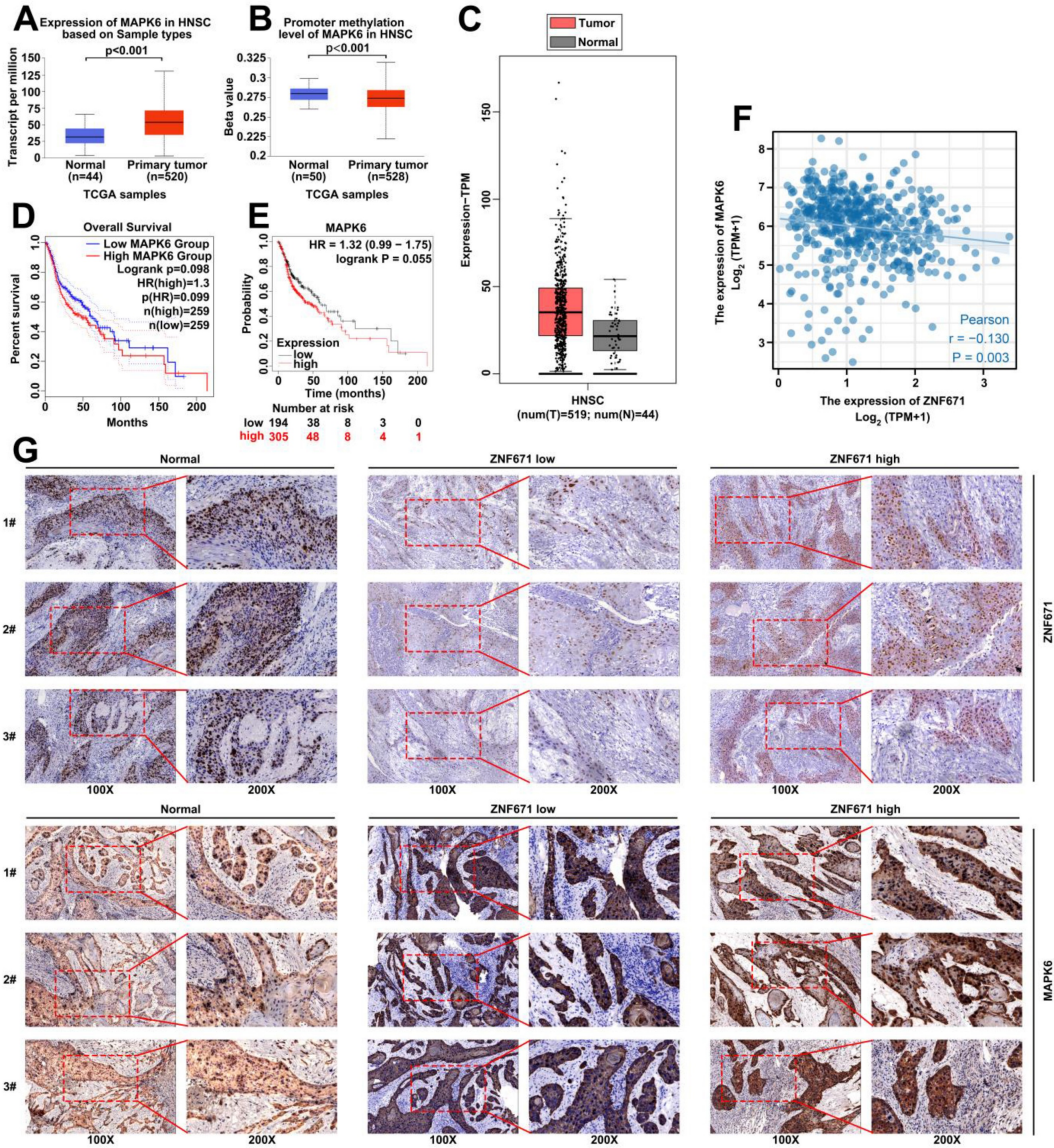
** MAPK6 is highly expressed in LSCC.** (A-C) The expression level of MAPK6 in head and neck cancer (TCGA-HNSC) was analyzed using online platforms UALCAN and GEPIA based on TCGA database. (D-E) GEPIA and Kaplan-Meier were used to analyze the effect of MAPK6 expression on prognosis. (F) The correlation between ZNF671 and MAPK6 expression was analyzed using TCGA-HNSC data. (G) Immunohistochemical analysis of MAPK6 expression in LSCC tissues with high and low ZNF671 group.

UALCAN and GEPIA were used to analyze the expression level of MAPK6 in TCGA-HNSC based on TCGA database. The results demonstrated that the expression level of MAPK6 was wincreased and the methylation level was decreased in HNSC (Fig. 6, A-C). GEPIA and Kaplan-Meier plotter were carried out to analyze the effect of MAPK6 expression on prognosis, and the results demonstrated that patients with high MAPK6 expression had poorer prognosis (Fig. 6, D-E). In addition, TCGA-HNSC data were carried out to analyze the correlation between ZNF671 and MAPK6 expression, and the results showed that ZNF671 and MAPK6 expression had a negative correlation in HNSC (r = -0.13, *p* = 0.003; Fig. 6, F). In addition, we divided LSCC patients into low ZNF671 group and high ZNF671 group according to the expression of ZNF671, and then analyzed the expression of MAPK6 in them by immunohistochemistry. We found that the expression level of MAPK6 in LSCC tissues was observably higher than that in the normal group. Especially, the expression was more obvious in the low ZNF671 group (Fig. 6, G).

### Up-regulation of ZNF671 inhibited tumor growth *in vivo*

**Figure 7:**
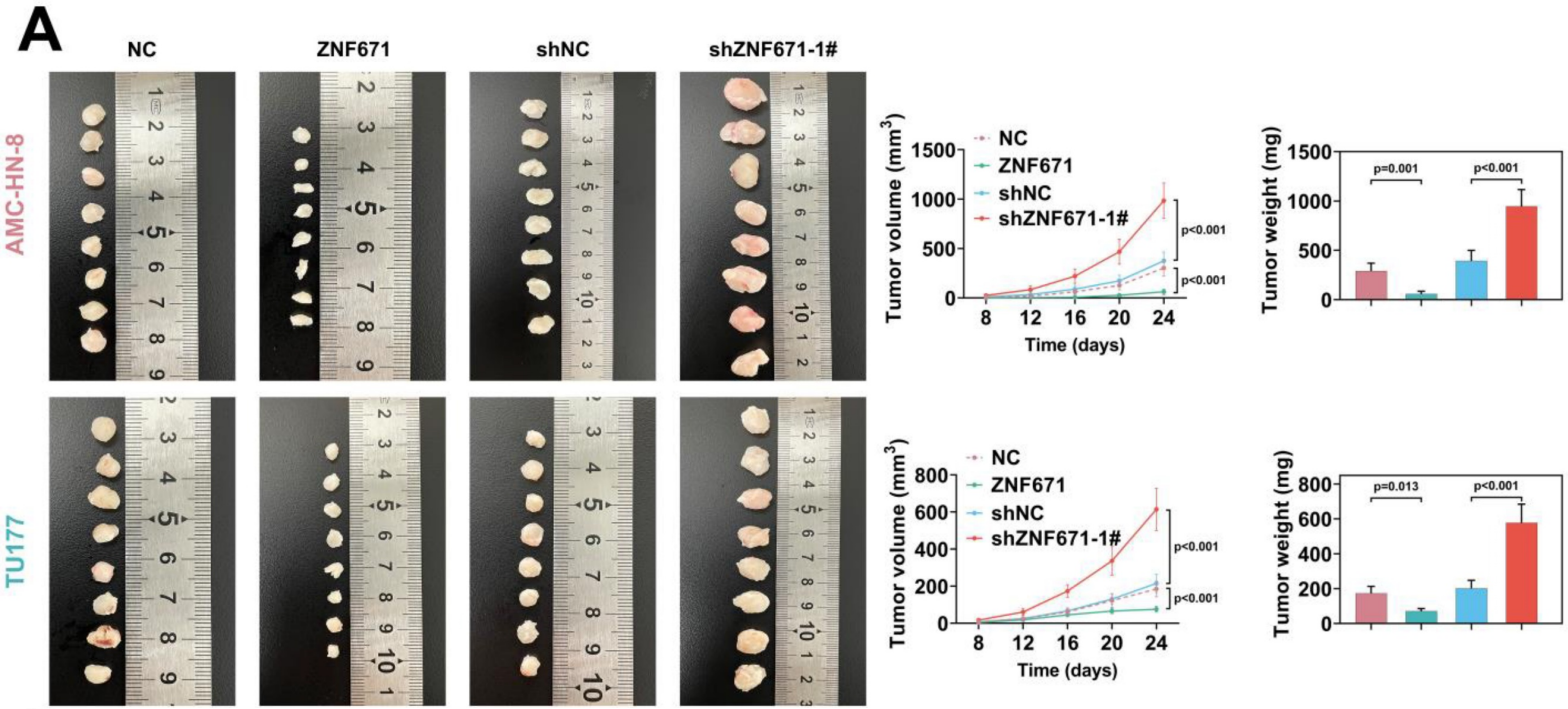

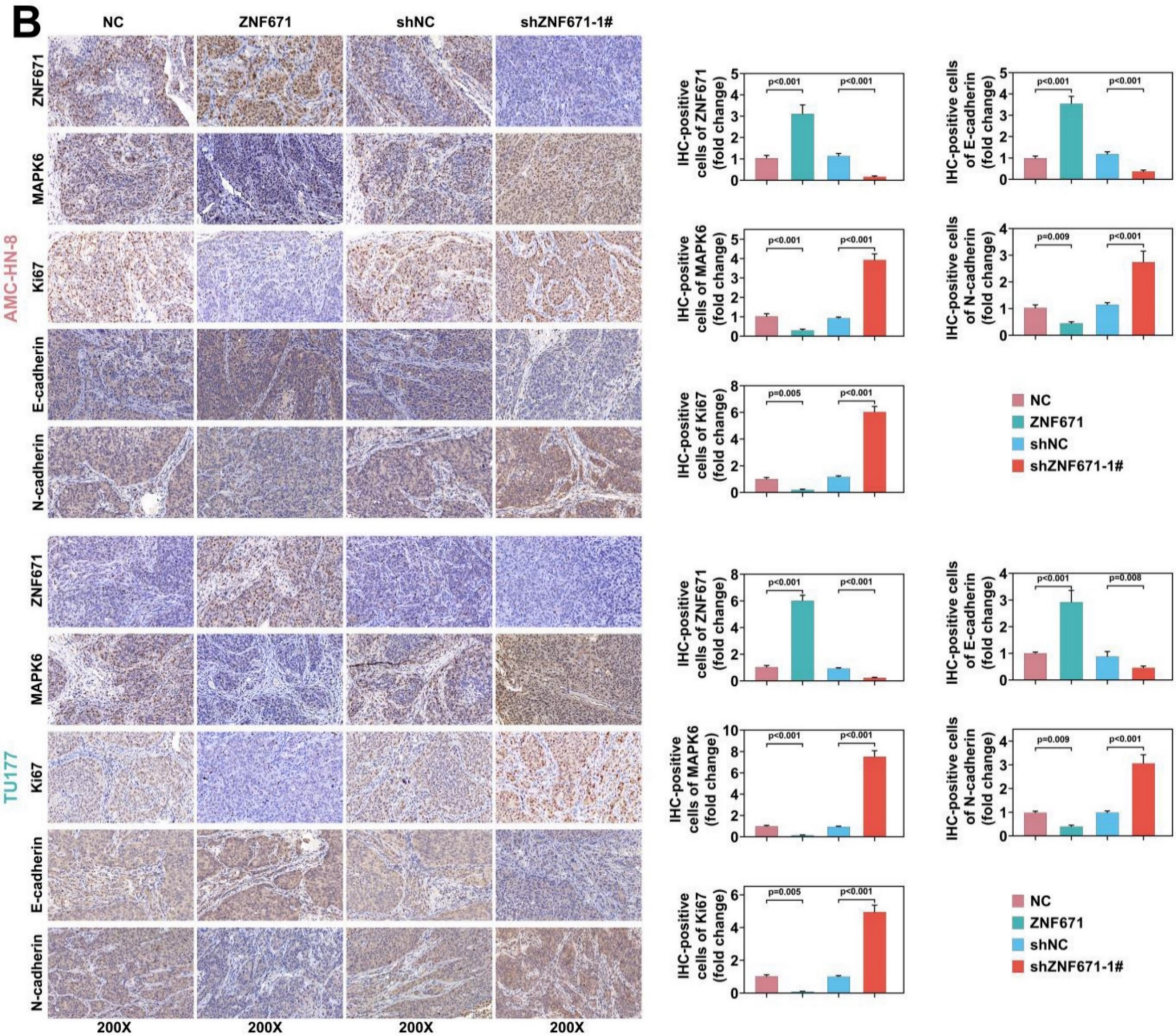
** Up-regulation of ZNF671 inhibited tumor growth and MAPK6 expression *in vivo*.** In BALB/c nude mice, AMC-HN-8 and TU177 cells overexpressing or knockdown ZNF671 were injected subcutaneously to analyze the tumor volume and mass (A), and the expression of related proteins was detected and quantified by immunohistochemistry (B).

AMC-HN-8 and TU177 cells overexpressing or knockdown ZNF671 were subcutaneously injected into BALB/c nude mice, and the tumor volume and weight were measured. The results unfolded that the tumor volume and weight were deceased in the ZNF671 overexpression group (Fig. 7, A). In addition, the expression of related proteins was detected and quantified by immunohistochemistry, and the results showed that overexpression of ZNF671 could inhibit Ki67, MAPK6 and N-cadherin, and enhance the expression level of E-cadherin (Fig. 7, B).

## Discussion

LSCC patients have a poor prognosis and poor quality of life after surgery 35, 36. Therefore, exploring the pathogenesis of LSCC is of great benefit for early diagnosis and treatment. In our study, 33 genes with high methylation level and low expression were identified by online database analysis, and we focused on ZNF671 among them. From them, we selected several genes to perform the further functional assays, and noticed that ZNF671 affected the progression of LSCC. Therefore, it has been identified. Through database analysis and clinical sample validation, ZNF671 was significantly decreased in LSCC. Functionally, ZNF671 inhibited the proliferation, migration and invasion of LSCC. Mechanistically, ZNF671 promotes MAPK6 expression by binding to its promoter region. *In vivo* experiments confirmed that overexpression of ZNF671 inhibited tumor growth.

Through the GEO database, we analyzed the genes with increased methylation level and decreased expression level, and obtained 33 genes, including ZNF671. Previous studies have demonstrated that ZNF671 is associated with a poorer prognosis of ovarian cancer patients 18. ZNF671 methylation is involved in the occurrence and development of various tumors 37, 38. We hypothesized that ZNF671 might affect the occurrence and development of LSCC. Further, the analysis of GEO database found that the expression level of ZNF671 in tumors was higher than that in the normal group, which was confirmed by our clinical samples. In addition, through correlation analysis, it was found that the low expression level of ZNF671 was closely related to the prognosis of tumor patients, that is, tumor patients with low expression level of ZNF671 had a poorer prognosis. Methylation specific PCR was applied to analyze the methylation level of ZNF671 in LSCC, and it was found that ZNF671 had a high methylation level in LSCC tissues, which was consistent with the low expression of ZNF671 gene.

To explore the mechanism of ZNF671 in LSCC, human laryngeal epithelial cells and LSCC cell lines were used. First, we verified that ZNF671 has a low expression level and a high methylation level in LSCC. To reveal the role of ZNF671 in LSCC, we overexpressed or knocked down ZNF671 in LSCC cell lines, and then found that ZNF671 could inhibit the proliferation, migration and invasion of LSCC cells by MTT, Edu, TUNEL, and transwell assays, while promote the apoptosis of LSCC cells. This is consistent with previous reports that ZNF671 is involved in tumorigenesis and development 17, 39.

As a member of the transcription factor family, ZNF671 can bind to the promoters of other gene and regulate the expression of other genes. By software analysis, we found that ZNF671 could bind to the promoter regions of MAPK6 and SIAH1. By overexpression or knockdown of ZNF671, it was found that ZNF671 could affect the expression of MAPK6 but not SIAH1. Therefore, we focused our attention on MAPK6. Previous reports have found that MAPK6 plays an important role in many tumors 40-43. We found that knockdown of ZNF671 up-regulated the expression level of MAPK6. ZNF671 could bind to the -2000 - -1500 and -1000 - -500 regions of MAPK6 promoter by fluorescence reporter gene and ChIP-PCR analysis.

Based on database analysis, we found that the expression of MAPK6 was increased in head and neck tumor tissues, and our clinical samples also confirmed that the expression of MAPK6 was increased in LSCC, and the high expression of MAPK6 was positively correlated with the poor prognosis of tumor patients. In addition, we also found that MAPK6 was highly expressed in tumor tissues, especially in the low ZNF671 group. Furthermore, *in vivo* experiments confirmed that overexpression of ZNF671 could inhibit tumor volume and weight, and down-regulate the expression of MAPK6.

Our study still has some limitations. For example, we did not investigate whether ZNF671 is related to other tumor characteristics, such as stem cell-like properties, epithelial-mesenchymal transition, etc.

In conclusion, our study found that ZNF671 expression was down-regulated in LSCC and was associated with poor prognosis. ZNF671 up-regulates the expression of MAPK6 by binding to its promoter region, thus participating in cell proliferation, migration and invasion in LSCC. Our study may provide new ideas for early prediction and treatment of LSCC.
